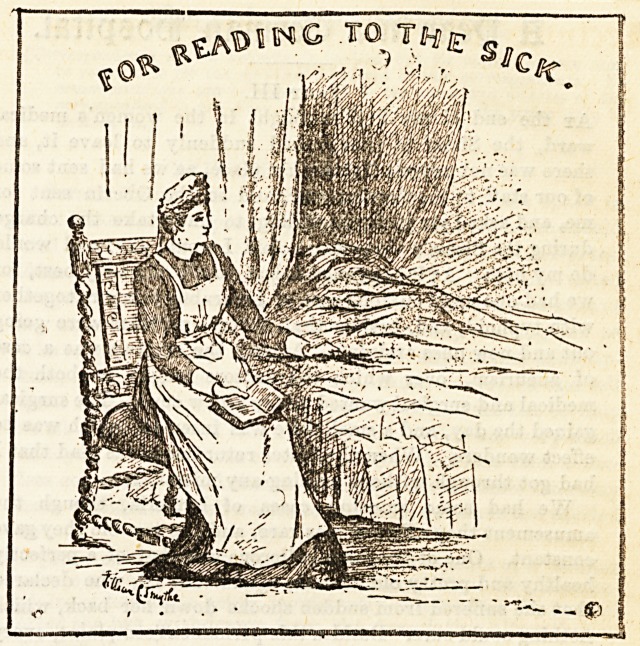# The Hospital Nursing Supplement

**Published:** 1891-06-20

**Authors:** 


### "The Hospital" Nursing Mirror.

Being the Extra Nursing Supplement of "The Hospital" Newspaper.

Contributions for this Supplement should be addressed to the Editor, The Hospital, 140, Strand, London, W.C., and should have the word "Nursing" plainly written in left-hand top corner of the envelope.

### En Passant.

THE BRASSEY HOLIDAY HOME.—We have received the following account of the first picnic of the season at St. Leonards: "The morning decided upon was most favourable, so we started off for Ecclesbourne Valley soon after ten a.m., with hampers packed full of good things. Arriving at Ecclesbourne about eleven a.m., our visitors had time for a ramble through the wood and glen whilst we prepared luncheon for one o'clock. They all returned by that time with sharpened appetites, and thoroughly enjoyed the repast. We met again at four o'clock for tea, which we had in the valley, and whilst we were seated round our tablecloth spread on the grass a saucy little chicken darted on to the table and made off with a sandwich. Every one made a grab at the little thing, but he got clear away, not before he had overturned the slop-basin, with all its contents, which greatly increased the fun. We started for home about half-past six, having had a most happy day. Our next picnic, we hope, will take place at Battle on the first Tuesday of next month."

THE SANTA CLAUS HOME.—There was a very pretty little ceremony at the opening of this Home for Children at South Grove, Highgate. The Home is only a small one, containing eight cots and a cradle. It is under the management of the Misses Charles, while Miss Napper is the Matron. The rooms are very bright, and all the cots are "named" already. Everything promises well for the success of the Home, not the least point being the fact that such an experienced matron will reign there. Lady Lucy Hicks-Beach proclaimed the Home open, and hoped it would prove beneficial to many children. A short service followed, and some appropriate hymns were sung. Only ten years ago it was almost impossible to find homes for children with spinal disease or abscesses, or any of the long tiresome illnesses which utterly ruin child-life, and can only be cured by long treatment in bracing air. But now the public is awake to the cry of the invalid, as well as the sick child, and every effort is being made to brighten these dullest of little lives. May we remind our readers that the Home would be glad of a piano as a gift?

THE NURSE-DOCTOR SYSTEM.—The question of a House-Surgeon for Walsall College Hospital is once more to the fore, and this time will probably be settled by the appointment being made. At a late meeting of the Committee, Mr. J. Leckie said the hospital, he was sorry to say, stunk amongst the workpeople who came there, and he appealed to the friends of the institution not to stick to Sister Dora traditions, seeing that her successors—worthy and devoted though they were—had not kept the hold upon the affections of the people which she had. Mr. Crump, on behalf of the committee, said they came with a free and open mind to the discussion of the question, and maintained that the charge that the patients were treated by unqualified assistants fell to the ground, giving in detail his grounds for holding that view, and adding that since an inquiry which was held in that room four or five years ago, the sisters had not been allowed to take any part in the surgical treatment of patients, and had been restricted to first aid. A sub-committee has been appointed to discover the opinion of the town, and see if the increased cost of a House Surgeon could be met.

ST. MARY ABBOTTS INFIRMARY.—A scheme for the training of probationers at the Kensington Infirmary has been accepted by the Local Government Board, which provides that at the expiration of three years such probationer, upon passing the prescribed examinations, will receive a certificate of nursing. They will be instructed in theoretical and practical nursing by the Medical Staff and Matron. It has been found that any period of training short of three years is unsatisfactory.

THE ADVANCE OF THE ATTENDANT.—The good news this week is from the City Asylum, Birmingham. In May several of the attendants and nurses went up for the examination of the Medico-Psychological Association, and the following have secured certificates: Nurses S. A. Devlin, F. Parkes, C. Coaling, M. Moon, M. Kings, E. Bearpark, A. Phillips, M. J. Moore, and M. Allbut; Attendants G. Evans, J. Parkes, W. Butterworth, T. Connor, and Head Attendant George Lees. Kings and Butterworth took their St. John's certificates last year.

SHORT ITEMS.—The nurses of the Kingston Infirmary have, by agitation, secured a half day off duty weekly, and a whole day monthly.—The Balloon Society has awarded its silver medal to Mrs. Grimwood.—Anyone who wishes for half-an-hour's gruesome reading had better secure the pamphlet issued by the Midwives' Institute—"Disastrous Results from the Practice of Uncertificated Midwives."—The Princess of Wales is getting up a subscription for Mrs. Grimwood.—The Chaplain of the Leper Hospital, Colombo, thanks those readers who last year sent scrap-books to him through Miss Gethen.—The Queen's Hospital, Birmingham, proposes to start a private nursing staff.—During May the Queen's nurses paid over 2,000 visits.—Mr. Wiggin, M.P., presided over the annual meeting of the Harborne District Nursing Society, and dwelt on the value of the services rendered by Nurse Williams.

DOMESTIC SERVICE.—It is strange that our pages should hold a controversy on the vexed question of mistresses and servants, and after this week we do not propose to proceed with an unprofitable discussion. It does not make matters better for mistresses and servants to revile one another in print. It does so happen in this life that good mistresses sometimes get bad servants, and that good servants get bad mistresses. But still, a little largeness of character, an attempt not to fret over trifles, but to be bright and good-humoured, and enjoy the give-and-take of life, can smooth over most disagreements. The public is being told just now that all nurses are angels, and that all domestic servants are the other thing. This is not so. Our Black List of unsatisfactory nurses grows day by day, whereas day by day we come across domestic servants who are so willing and hardworking that they make the households they serve go smoothly and cheerfully from week's end to week's end. Let the servant have patience if the mistress is suspicious and petty, and in time the mistress may become trustful and kind; and let the mistress care for her servants, and let them have fresh air and wholesome surroundings, and a nice bookshelf of books to read when they have an hour off duty. The good woman, no matter what her station in life, knows how to bear and forbear.

### Lectures on Surgical Ward Work and Nursing.

By Alexander Miles, M.B. (Edin.), C.M., F.R.C.S.E.

### Lecture XXVII.—BANDAGING.

It is scarcely necessary that I should indicate in any but the briefest manner the numerous uses to which the bandage is put in surgery. All dressings, fomentations, and compresses are fixes and retained in position by one or other form of bandage ; and almost all splints, whether applied to control fractured bones, to ensure immobility of joints, or to correct deformities, are similarly secured. Bandages, too, are often used alone, or impregnated with various substances, such as starch and plaster-of-Paris, to give rest or stability to different parts. As a means of preventing and of arresting hæmorrhage, and of influencing the size of certain forms of swelling also, different varieties of bandage are of great value.

Materials.—The variety in uses to which bandages are put is only exceeded by the materials from which they are made. (1) Linen, cotton, and calico form useful, strong bandages, varying in strength and stiffness according to the quality and newness of the material. They are softest and lie best when made of old stuff which has been frequently washed. These bandages are not antiseptic, and must, therefore, never be used in dressing open wounds, or in applying antiseptic fomentations or poultices to an unopened abscess. (2) Gauze. This material is usually charged with some antiseptic agent, and so can be employed with safety in dressing aseptic wounds. Sir Joseph Lister first used gauze impregnated with carbolic acid, and later with the double cyanide of zinc and mercury. (3) Domette is a variety of soft, elastic flannel, and bandages made of this material fit nicely to the part, and do not tend to slip, as stiffer forms sometimes do. They are usually rendered antiseptic by being charged with Sal Alembroth, and as they wash well may be used repeatedly so long as they are always reimpregnated with the antiseptic. (4) Muslin, strong and course, with wide meshes, is used in making plaster-of-Paris bandages, the dry plaster being rubbed into the muslin and filling up the meshes. (5) Elastic webbing is a form of bandage in which the longitudinal strands are made of elastic, the transverse ones being cotton. Such a bandage is used to apply a moderate amount of pressure or elastic support to any part; for example, a joint into which there has been some effusion, or in a case of varicose veins. The open network has the advantage over simple elastic bandage of permitting evaporation of the sweat, which, if retained, irritates the skin. (6) Martin's Elastic Bandage is made of a sheet of thin elastic. It is chiefly used in the treatment of varicose veins of the leg, but, as just mentioned, is objectionable in retaining perspiration. In cases of emergency old household linen (short of being rotten—the older the better) makes very good bandages.

Varieties of Bandages.—The commonest form of bandage used in surgery consists of a length of material rolled tightly up from end to end, constituting a simple "roller bandage." When rolled from one end only it is spoken of as a "single-headed" roller bandage, when from both ends as "double-headed." It will be necessary later to describe certain forms of compound bandage, such as the many-tailed, four-tailed, T-shaped, and so on, each having a special use. Again, names have been invented to indicate the various methods of applying a bandage to a part, depending on the arrangement of the turns of the bandage. For example, a "circular" bandage is one in which each succeeding turn exactly covers the one which preceded it; an "oblique" bandage simply takes its own course round the limb, covering parts, and leaving others bare, a "spiral" bandage, evidently from its name, winds spirally rounds the limb, but each succeeding turn overlaps only two-thirds of its predecessor. An important modification of the last is the "spinal bandage with reverses," in which the bandage is folded over on itself at regular intervals, to ensure that no spaces will be left between the turns uncovered. The "figure of eight bandage," as its name implies, consists of two almost equal circular turns, one being above, the other below the point of crossing. It is chiefly employed in covering in joints. Closely related to it is the "spica bandage," so named from a fancy resemblance to a spike of barley. In it one circle is larger than the other, it being employed in bandaging parts where two unequal cones meet, as for example at the shoulder, or groin. The indications for selecting one or other of these forms will be pointed out later on, as well as the means of carrying out the manipulations.

Parts of a Bandage.—For purposes of description, various names are applied to different parts of a bandage. Thus the part of a roller bandage which is still unrolled is spoken of as the *head*, and naturally the loose end is referred to as the *tail*. The *anterior surface* is that on which the head rests, while of course the opposite side is *posterior*. As the bandage is being applied to a limb, the edge nearest the trunk of the body, that is, highest up the limb, is called the *upper margin*, and the other the *lower margin*. Such names are not necessary when a practical demonstration of bandaging is being given, but they facilitate a word description of the appropriate manipulations.

Sizes.—It is obvious that different widths of bandage will be necessary for different parts of the body, bearing some proportion to the circumference of the part to which they are applied. The sizes usually recognised are : For upper extremities and head, 2½ in. wide; for lower extremities and pelvis; 3 to 3½ in. wide; for thorax and abdomen 4 to 4½ in. wide. These are respectively known as "sixteens," "twelves," and "eights," because from a piece of calico one yard wide sixteen bandages of the first size, twelve of the second, and eight of the third can be obtained. They should all be six yards in length.

To Make Bandages.—In their surgical handbook, Messrs. Caird and Cathcart describe the process briefly as follows : "Procure six yards of calico, about one yard in width, and remove the selvedges. Mark off with scissors short strips of the desired breadth; then grasp the alternative strips gathered in two bundles and pull in opposite directions." In this way a number of strips can be rapidly and evenly torn, and must then be rolled. This may be done either with the hand or with a machine made for the purpose, the main objects being to have the bandage tight and evenly rolled. When using the bandage machine some difficulty will be experienced in withdrawing the pin from the centre of the bandage unless the first few turns be made rather slack, and the handle reversed once or twice while the completed roll is firmly grasped by the hand. Tie a few threads from the edge of the bandage round the roll to prevent it coming undone.

The Parts to be Bandaged.—The exact variety of bandage to be used in any given case depends entirely on the shape of the part to be covered in, and if we consider the shape of any segment of the body we shall find that it is either a cone or part of a cone, or else is made up of the junction of two cones. Here and there short areas, more or less cylindrical, are to be met with, but these soon become conical. For example, take the upper extremity. From the finger tips to the middle of the palm of the hand we have a slight cone, the base being at the latter level, and from this starts another with its apex at the wrist. Just above the wrist we meet with a short cylinder, which, however, soon expands into the cone of the forearm. At the elbow joint this cone meets that of the upper arm, giving us a well marked junction of cones. And so on all over the body, the lower extremity, the trunk, and even the head and neck being each capable of resolution into these geometrical forms. For cylinders, then, we use the ordinary spiral bandage; for cones, the reversed spiral; and for the junctions of cones the figure of eight, or the spica.

### Women as County Councillors.

AT an interesting meeting at the Rev. and Mrs. Haweis's house, on May 30th, this subject was fully discussed, especially in so far as it bears on the care of the sick and the insane.

Lady Sandhurst gave a most interesting address, giving her own experiences, and urged strongly the duty that women owed to the helpless of both sexes, as well as the children, whose lots in life were determined by the decision of the County Council, and spoke feelingly of the truly womanly nature of the work. Miss Cobden supported her, and mentioned the need of women visitors to the female wards of lunatic asylums. All the speakers spoke very warmly of the value of Miss Cons (the lady Alderman), who was one of the unwilling absentees from illness.

Another important point was alluded to. Should the schools and workhouses be taken over as part of what was justly described as the domestic parliament of the nation, and the present strange anomaly continue that women might legally stand, be elected, and yet not give their votes? What would become of our invaluable lady guardians and members of the school boards?

One speaker mentioned how feminine influence was needed in the boys' industrial schools, leaving it to be inferred how much more it must be needed in girls' schools.

Perhaps one of the most important points was hardly touched on—the question of the appointment to posts of matrons of any institution. If it is considered for a moment the care that is given to testing the character of any servant in a well-regulated family, to the daily ordering of the household routine, to the details of nursery management, to the early education, clothing, and health of young children, to the care of the sick and aged in all households; and then inquire, who is it that does these important duties? Is it the husband and father, or the wife and mother? and failing her, does not the daughter take her place? Does any woman know any well-ordered household where these functions fall to the lot of the men of the family? and if it is so in daily life, where there can be but little selection, will not it be much more so where the woman is chosen specially for her aptitude for this work, not by the one man who is in love with her, and would rather have her misrule his household than have any one else govern it well, but by keen-sighted ratepayers, men and women who know that it is their money that is being spent, and who, no doubt, also feel that it is their duties to the young, the sick, and the insane that they are delegating, and that it is no light responsibility that is undertaken, and that far from throwing obstacles in the way of such women as are willing to undertake these onerous duties, all men should do everything in their power to smooth the difficult path, and to welcome as many fit and capable women as can be persuaded to devote themselves to the good of the nation, and the care of those who are dependent either from age, sickness, or the sins or misfortunes of others, on the care of such bodies as Boards of Guardians or County Councils.

### Appointments.

FUSEHILL HOSPITAL.—Nurse Toft, who for many years has been a nurse at the Withington Workhouse Hospital, was on Friday last appointed Assistant Nurse at the Fusehill Workhouse Hospital, Carlisle.

MISS MINNIE DRAKARD has been appointed Sister of the Typhoid Fever House at the General Hospital, Nottingham. Miss Drakard was trained at the Boston Hospital, Lincolnshire, for three years, and has been Staff Nurse in the General Hospital, Notts, for two years.

### WHERE ARE YOU GOING?

THIS is not a question everybody can answer off hand, for a great many never think of putting it to themselves at all. Still, it is very necessary to do so, lest we be taking the wrong road ignorantly.

In old times there was a common saying, "All roads lead to Rome." It was then the capital of the world, the place where every one longed to go, and which the energetic, from far or near, strove their utmost to reach, and having got there, remained for the rest of their lives.

Now, we have "an abiding city" offered to us, where everything rich and rare is stored for our benefit, fair clothes and golden crowns, streets of gold with pearl gates, and all sorts of delicious fruits for our food. There the sun always shines and refreshing streams flow for our use. There is no sorrow nor sighing in that beautiful home, all tears are wiped from every eye, and death can never part us from those we love. You have heard of that place as Heaven, and though we all hope to gain it in the end, are we turning in the right direction now? Where are we going? Not with the foolish ones of the earth, let us hope, who follow the broad road to destruction, which is so easy to find and seems at first so full of flowers; but they speedily turn to thorns, which tear our flesh and hurt us grievously. No, "things are not always what they seem." Idleness is pleasant, but go by the garden of the sluggard, and we plainly see "the thorn and the thistle grow broader and higher," and the person, be it man or woman, who flies to the wine cup for delight and solace finds ruin and misery in the bitter dregs.

As these, then, are not the paths in which we should tread let us go to that guide which never fails us, the Bible. There we are told by Christ that the way to everlasting life in the mansions which He has "prepared for them that love Him," is a narrow and difficult one. There is no mistake about it, whatever is worth having costs toil and self-denial, frequently more than we are inclined for, sometimes more than we can do. But we have always One to fly to in all trials, Who is ready to take our burdens upon Him if we seek first the Kingdom of God and His righteousness.

We must put God first, His love, His laws, and His commandments, and then all good things shall be added to us. No longer will we try blindly to find our own paths, but commit our way to Him "whose ways are ways of pleasantness and all His paths are peace."

Lead, kindly light, amid the encircling gloom
Lead Thou me on;
The night is dark, and I am far from home,
Lead Thou me on.
Keep Thou my feet; I do not ask to see
The distant scene; one step enough for me.
I was not ever thus, nor prayed that Thou
Should'st lead me on;
I loved to choose and see my path; but now
Lead Thou me on.

### A Year in a German Hospital.

Part III.

At the end of my first fortnight in the women's medical ward, the Sister in charge had suddenly to leave it, and there was not anyone to take her place, as we had sent some of our staff to the Servian war, and so the Oberin sent for me, and asked me if I was willing to undertake the charge during the Sister's absence. I said I would try, and would do my best. As it happened I had to do my very best, for we had one most anxious case of incurable tumour, together with typhoid, and, besides that, several patients were going out and new ones arriving. Among the arrivals was a case of aneurism, over which the doctors fought, as both the medical and surgical wanted to try a new cure. The surgical gained the day, and a small wire was inserted, which was to effect wonders. When the Sister returned I was glad that I had got through without making any mistakes.

We had some amusing cases of hysteria, though the amusement they afforded was rare, and the trouble they gave constant. One of the most ludicrous was that of a perfectly healthy and pretty old woman, aged about 60, who declared that she suffered from sudden shocks down her back, which nothing could cure. Besides this permanent complaint, every few days she developed a new symptom, and one week the shocks took to coming in her teeth, of which she had very few. She said (contrary to the statement of the Sister who had had night duty) that she had not been able to sleep or eat in consequence, so the doctor said he would pull out the worst one that afternoon. This was not at all what she wanted or had expected, and when the time came and he brought a case of implements, and looked into her mouth, out the old lady sprang on the other side of the bed and ran half way down the ward in a very short night garment, with bare feet, and her white hair flowing. The doctor tried to look and be severe, ordering her back to bed, but she had to be caught and held during the performance. She behaved like a child of three, struggling, screaming, and addressing the doctor as a naughty boy. Finally, she did not know when it was out, and consequently when we let go was out of bed again, declaring she would not have it done. It so cured her malady that she was well enough to leave the next day. Another woman who was the unconscious cause of general mirth was brought in one night stiff and rigid. With much care and difficulty we undressed her, sending at once for the doctor. He tried to rouse her, with such good effect that she sprang up, clasped him tightly round the neck, called him "her darling Fritz," begged his forgiveness, "as she would never do it again," and besought him to go to bed at once. The doctor was young, and got very red and angry, the nurses having much ado not to laugh till he was gone. The next morning the woman went out much ashamed.

The next ward I worked in was the women's surgical; I attended any number of operations, and learnt a great deal. It was Christmas time, and that means a great deal to the Germans. The Empress gave a certain yearly sum to be spent only on the patients at Christmas; to each Sister was apportioned a share for her ward. I went to cater for the children one day at the large wholesale shops, and bought every toy that can delight girls and boys from the age of one to twelve years. We brought them all home in a "droschke;" when the door was opened we were not to be seen at all, and until the parcels gradually fell out we could not move.

A Christmas-tree was prepared and decorated for each ward, and kept very secret and out of sight. The wards were made perfectly lovely with flags, flowers, and bright things. One Sister made designs out of dried thistles, which were most artistic and effective.

Besides all the presents and preparations in the hospital, each Sister bought or made clothes for some poor family outside, generally one which had come under her notice in the hospital. On Christmas morning, as early as possible (we went about seven o'clock), we carried all we had collected, in the way of clothes, food, sweets, and toys, in a large carpet bag to our family; we had a small tree which we lighted up before the door, and then brought everything in, singing a hymn as we came. We dressed the mother and children in some of the garments, unpacked the bag, and then left them to enjoy themselves. The woman kissed the Sister's hands, and the tears prevented her saying how grateful she was. It was certainly the happiest way of beginning the day to the givers. Each Sister had some poor family, or old and lonely man or woman whom she visited and made happier in this way on Christmas Day. The Empress came to chapel in the hospital on Christmas Eve, and after a nice service she and her suite adjourned to the conference room, where a large tree was brilliantly lighted, and hung with glass icicles, and the long table loaded with presents. At the Empress's request, each Sister was presented with a new habit, the Oberin with a writing-case, and the professors with books; she talked to them some time, and asked the professors questions; she then went into the servants' hall, where another tree and table were set out. All the men and maids were dressed in their best, and standing round they certainly did look just like brooms propped against the wall —but it meant loyalty. They all received some present, and the choir Sisters sang a very pretty "chorale" in parts. The Kaiserin then went into the ward-maids' hall, and the same ceremony took place; and after that we had supper, which we wanted very badly.

The next night the presents were to be distributed in the wards. All the ladies and gentlemen who were on the Committee, or were interested in the hospital cause, also the doctors and professors, the Oberin, all the Sisters and the lady pupils. We began with the men's medical ward; a large tree was raised high in the middle, all round it being ranged the presents given by the Empress and others. Over each bed was a text and a candle; in one or two wards was a manger made and arranged by the patients. The Chaplain gave a very short and bright address, the ward-men sang, and then the choir Sisters. All the presents were then brought to those in bed, and everyone talked to them to amuse them for a few minutes. All those who had been in the hospital for the whole year were given some really nice present, such as a watch, or warm clothes, or money. This was repeated in each ward, and it was rather late before we got to the last, but it seemed much shorter than it really was, as every one tried to make others happy. The merriment was so simple and natural, it was as if the poor things could not help themselves. I have never felt the same in England; the merrymaking always seems rather forced and boisterous at Christmas time. The children's ward was presented with a musical-box and a doll's theatre, the other wards with an easy chair, or some kind of extra convenience. I have not mentioned eating and drinking, but, of course, that plays a very large part in the festival, much larger than in England, only there is so little drunkenness in Germany, as they are so accustomed to beer that they can stand a great deal. The New Year was also celebrated as a great festival, with much singing and eating, but I did not see so much of it, as I was then nursing the first and second-class patients. After my month with them I took night duty in the whole of the women's wards, medical and surgical, for six weeks, and consequently I had a great deal of responsibility, and less hard work. At the end of that time my year was over, and I went for a rest, having learnt to love the Sisters and many of the patients, and to find great happiness in working hard, and thinking about other people.

### Nurse Training in the Seventies.

BY E. D., Author of "Recollections of a Nurse."

*(Continued from page xlvi.)*

No nurse could give an exact account of a day or night's work in hospital, the hundred and one things that have to be done requiring hands and head, questions asked well nigh impossible to answer, sleepless patients and fretful ones to be soothed, poultices, fomentations, and liniments to be applied, so that, whether on day or night duty, the nurse's or probationer's work is incessant, and this not in good, fresh air, where you might expect an appetite which requires no coaxing, but at best in air which is loaded with the smell of disinfectants. Therefore, a nurse's food should be cooked and served up in an appetising form. The breakfast should be a good, nourishing one—well-boiled oatmeal porridge, with either fish, bacon, or eggs. When fish is cheap, why should not a change be made to a plentiful supply of redgeree, with tea and coffee? Instead of beer and bread and cheese at eleven o'clock, I would give them a cup of milk and a whole meal biscuit. Then for dinner a good meal of freshly cooked butcher's meat, with two kinds of vegetables. Tea, bread and butter. If fruit can be got at a reasonable price, give it them. The suppers have generally been a trial to most matrons to get a variety which shall be inexpensive. In hot weather nurses are always satisfied with cold meat and salad, milk pudding, and sometimes stewed fruit. But in carving for the mid-day meal let it be so done that a whole joint be left if possible for the supper. This is far more appetising than two or three joints spread about the table with pieces hanging to the bone. It is only a matter of deftness in carving. Salads should be fresh and crisp, not a dish of faded leaves; so many different kinds can be arranged from cold vegetables left from the mid-day dinner, only let the things be fresh and clean. Then there are various winter supper dishes, the well-known potato pie, which I saw in a nursing home last winter, both juicy and savoury. Any day the meat happens to be underdone near the bone for dinner cut it in small square pieces and bake in batter. Or if boiled veal or mutton should be the dinner, nothing makes better curry of already cooked meat, or grind up the meat and put equal parts of boiled rice or bread crumbs, and fry as rissoles. Most hospitals give their nurses boiled salt beef once a week. Why should not the beef be fresh? Then the water it has been boiled in can be used for lentil soup, with the addition of carrots, turnips, and onions; then the good old-fashioned mutton broth with suet dumplings and pearl barley. Then an ordinary baked suet pudding with a fair quantity of finely-chopped onions and parsley, pepper and salt, is often liked. Sardines fried in batter is another thing. Macaroni stewed in stock and grated cheese, with pepper and salt, tossed in, serve it very hot in a pie dish, or ground Indian meal well boiled and poured into dishes an inch deep, well sprinkled with grated cheese, and bake, besides numberless other things; and occasionally some well-disposed landowner whose rabbits are a nuisance to him would find a dozen or so would not be to the matron of a London hospital. They must be careful with the money entrusted to them by the public. They may do this and still give plain, wholesome food to the nursing staff, and they should all have their meals together and share alike. No woman should think of taking up a nurse's life unless she is capable of some self-sacrifices. The cry for some years has been, Give us a better class of women, more refined, better educated. Are these women prepared to give up the fleshpots of Egypt, to be content with plainly-cooked food, to be, in fact, a help to the suffering poor, to forget self in their work? Only such women should be accepted in any hospital; only such women are worthy of the name of nurse.

### Everybody's Opinion.

*[Correspondence on all subjects is invited, but we cannot in any way be responsible for the opinions expressed by our correspondents. No communications can be entertained if the name and address of the correspondent is not given, or unless one side of the paper only be written on.]*

### BRITISH NURSES AGAIN.

SISTER FROST writes: Will you kindly allow me space in THE HOSPITAL to express my disapproval that my name should still continue to be on the Council of the Royal British Nurses' Asssociation. I enclose you the first and only receipt dated August 13th, 1889, and a few days after this I wrote to have my name withdrawn. I never attended any meeting and as I have not had any notice asking for any further subscription, naturally thought that my name was erased and self quite forgotten. Not so; my name is still on the list to help swell the number of the R. B. N. A., and I am very much annoyed to think I was so weak as to be led into such a useless concern. I would like to warn my fellow nurses who may have withdrawn their names to see their demands are carried out.

[This substantiates various other statements of a similar nature we have received; for instance, Mrs. Franks complains of her name being persistently retained on the council after she has withdrawn from the Association altogether.—EDITOR.]

### THE DULNESS OF DOMESTIC SERVICE.

"MARY" writes:—I have been in service over twenty years, and beg to state servants are hired to work now, or slave, as well as thirty years ago. It is quite necessary to ask the mistress questions before engaging to fill a situation, or you soon get told, "You did not object at first, now I expect you to do all I ask you." "A. E. S." is very liberal with wages, if her servants can afford to go shopping two or three times a week. I could not get out once a quarter without being told I was always wanting to go out, and then get grumbled at for leaving washing-up to do when I came in. It was a family of eight, and two poor slaves kept, who were called "my" cook and "my" housemaid before visitors, but "tiresome girls" behind visitors' backs. We did not have time to think of Bank holidays, regattas, circus, concerts, theatres, nor picnics, for ourselves; but we always knew when the young ladies went, as we were often expected to sit up till they came in. Eye service, with many servants, is considered an abominable thing; for my own part, I can, and do, work much better when not being watched. I have gone without my meals to work, or my day's work would not have been more than half done. If a mistress is really conscientious, and gets hold of a respectable servant, I venture to say in nine out of ten cases her servant will be faithful and love her. Mistresses, quite as much as servants, make a house heaven or the other place. If they are simply required to do the work, how often do they have to do it with half the number of things required? No brooms, no dustpan, no basins, no dusters, no water-cans, no saucepans fit to use, no kitchen cloths, no pie dishes, no plates, unless they are washed between meat and pudding. One mistress did not give me a character; as she promised me a bad one, I did not apply to her at all. Since then I have obtained seven excellent characters from people who know how to treat servants, not as slaves, but as a faithful servant should be treated. I am now parish nurse in my own parish.

### Wants and Workers.

[Under this heading, we propose to try for a few weeks whether we can be useful to our readers in making the wants of some known to others who are willing to do what work they can to aid the great cause of curing and cheering the sick.]

*Acknowledgments.*—Papers have been received at 23 Ward, St. Pancras, with thanks. Ladies' belt and old linen have been received at Skipton, with thanks. Papers have been received at the Nurses' Co-operation, with thanks.

*Time Offered.*—In answer to your request for workers, I shall be very glad to be useful to any Secretary needing help in writing. I have also a copy of THE HOSPITAL I could send on monthly to any institution. I can also do plain knitting.—*R. P.*

### Off Duty

### One Evening.

"Earth hath no sorrow that Heaven cannot heal."—*T. Hood.*

The clocks of London had struck eight.

There was silence in the ward. It had been a long and heavy day, but the work and bustle were over now. The patients had been made comfortable for the night; the gas was lowered; and prayers had been read—prayers closing with the pathetic petition which has a deeper meaning here than when used elsewhere: "By Thy great mercy defend us from all perils and dangers of this night." Then came Sister's kind voice, "Good night, all." "Good night, Sister," and no further sound is heard in the ward. The tired nurse sits down at a table in the centre of the ward, where her quick ear can catch the first sound of pain, or call for her help.

A tired woman! who nevertheless calls for no pity from us, but who should rather excite the envy of those (their name in London alone is legion) who also sit down tired after a fatiguing day, with none of the happiness felt by one who has the consciousness that her toil has been in the service and for the good of others only. They, seeking to please self, have pleased no one, themselves the least of all. "We lose what on ourselves we spend." We keep for our own alone that which we spend freely on others.

The nurse is "on duty" still, and in a few minutes rises quietly, and goes to one of the beds, where a woman has begun once more, after a short doze, to toss restlessly about, and to talk rapidly in a low voice.

"You must try to be quiet, Mrs. Norton; you will soon sleep; you must try not to disturb the others."

The pillows were shaken up once more, and the curtains moved to keep the light from the too-wakeful eyes.

"Oh, if I could only go home—if I could only see how Tommy is," she moaned. "There's no one to look after him now his father has gone to work. He's the last one I had, and I know I shall never go out of here. Mrs. Brown promised sure she would bring him in every visiting day, and now she's not been near me for a week. Oh, I wish I could go out, Nurse."

Nurse, in her own quiet way, tried to soothe the desolate mother, promising her she would try to send for Tommy to-morrow, bidding her try to sleep, and then she would feel all the better when Tommy came, looking the while with compassion on the poor, wasted form of the woman, knowing that if Tommy did not soon come he would see his mother no more in this world.

The ward was silent once more for a few minutes. Then came the familiar sound of tramping feet of men carrying a new case on the stretcher along the corridor, and the door opened to admit a casualty from the surgery.

"Child—run over. Doctor sent word he would be here directly."

Nurse, with a quickness and energy which seemed to belong more to the beginning of a day's work than the end of one, undressed the boy and placed him in the cot before the House Surgeon appeared.

On examination the case proved a much more serious one than was at first supposed. The wounds were dressed, instructions given for treatment during the night, and the Nurse sat down by the cot to watch the child until the night nurse came on duty.

The silence was broken only by the cries of the child: "Where's my *muvver*. I want my muvver. I want to go home," and by the moans of the poor dying woman who, partly unconscious, repeated again and again, "They might have brought him; she promised sure she'd bring him."

The voice became fainter, until it was only a whisper; and Nurse, who had gone for a moment to fetch a drink for a patient, noticed she was quiet.

"Asleep at last, poor soul," she thought, as she went up once more with her shaded lamp to look at her. "I am glad she is asleep."

One glance nearer told the Nurse that she was asleep at last, indeed: in that deep sleep in which all earth's troubles are quieted.

"I wish Tommy could have come in time," Nurse sighed to herself, as she moved back to the cot-side once more.

There all her attention was needed, for the little life so badly bruised was flickering to its close, and the cry, "I want my muvver," was scarcely heard.

The clocks of London chimed out nine.

There was silence in the ward again.

Tommy and his mother had met at last!

"Dagmar."

### Presentation.

Miss Mona S. Mountford, on leaving the City Asylum, Birmingham, for her new post at Haydock Lodge, was presented by the officers and nurses with a fitted travelling bag and umbrella. Miss Mountford had held the post of head nurse at the asylum for four and a-half years.

### Notes and Queries.

### Queries.

(16) "Where can a consumptive patient be sent to for change of air free of charge, or could; perhaps, pay 1s. 6d. or 2s. a-week?"

(17) Could any of your readers kindly tell me where I could get an invalid carriage and horse to take a poor bedridden woman to her new home a little way out of town. A small payment only could be made.

### Answers.

*Ex-Nurse.*—No; not if the typhoid left no chronic weakness behind. Try a country hospital, where the work is lighter and the air purer than in the London hospitals. You began too young; no wonder you got typhoid.

*Nurse Gertrude.*—Get "Our Baby," by Mrs. Hewer, published by Wright, of Bristol, price 1s. 6d. The Secretary of the Queen's Nurses receives letters at St. Katherine's Hospital, Regent's Park, N.

*M. Pickford.*—We have sent your letter on to the proper authorities. You ought to be able to get The Hospital at all bookstalls. Thank you for letting us know.

*E. D.*—We have asked two lawyers, and they both say you cannot claim any fee for giving evidence at the inquest.

### Amusements and Relaxation.

**SPECIAL NOTICE TO CORRESPONDENTS.**

**Second Quarterly Word Competition commenced April 4th, ends June 27th, 1891.**

Competitors can enter for *all* quarterly competitions, but no competitor can take more than *one first prize* or *two* prizes of any kind during the year.

The words for dissection for this, the TWELFTH week of the quarter, being

"TRIPOS."

| Names. | June 11th. | Totals. | Names. | June 11th. | Totals. |
|---|---|---|---|---|---|
| Christie | 17 | 380 | Ivanhoe | 14 | 326 |
| Patience | — | 214 | Weta | — | 147 |
| Agamemnon | — | — | Lady Betty | — | — |
| Hope | 18 | 385 | Mortal | — | 76 |
| Reldas | 18 | 385 | Little Eliza | — | — |
| Lightowlers | 18 | 356 | Dove | — | — |
| Nurse J. S. | 19 | 329 | Ladybird | — | — |
| Qu'appelle | — | 170 | Psyche | 18 | 334 |
| Jenny Wren | 17 | 310 | Ugug | — | 229 |
| Wyameris | 15 | 384 | Harrie | — | — |
| Paignton | 16 | 322 | Grannie | 13 | 317 |
| Theta | — | 206 | Eale | — | 169 |
| Success | — | 17 | Grimalkin | — | 53 |
| Tired | — | 136 | Nurse G. P. | 8 | 109 |
| M. G. | — | 188 | | | |

### Notice to Correspondents.

N.B.—All letters referring to this page which do not arrive at **140, Strand, London, W.C.**, by the *first post on Thursdays*, and are not addressed PRIZE EDITOR, will in future be disqualified and disregarded.

## Figures and Tables

**Figure f1:**